# On the Value of Gastrotomy and Gastro-hysterotomy in Obstetrics

**Published:** 1841-03-13

**Authors:** Jos. Warrington


// MEDICAL EXAMINER.

DEVOTED TO MEDICINE, SURGERY, AND THE COLLATERAL SCIENCES.

No. 11.] PHILADELPHIA, SATURDAY, MARCH 13, 1841. [Vol. IV.

### ORIGINAL COMMUNICATIONS.

### *On the Value of Gastrotomy and Gastro-hysterotomy in Obstetrics.* By Jos. Warrington, M. D.

Of the various resources within the reach of the accoucheur in the practice of the art of delivery, that which involves an extensive incision into the cavities of the abdomen and uterus, with a view to remove thence a child which could not, without the certainty of its own destruction, and in many cases the imminent risk of the mother, be extracted through the natural passages, appears to have been thought of, and under certain circumstances acted upon, from a period almost coeval with the history of the race of man.

Without stopping to inquire, whether Mercury did, as is said, extract the son of Jupiter from the belly of Semele—whether Esculapius was delivered in the same way by the surgical skill of Apollo—whether the operation has been entitled the Cæsarean operation, because one of the Cæsars was removed from the abdomen and uterus of his mother by gastro-hysterotomy, or whether the name of Cæsar was given to the subject of this mode of delivery, we propose to pass almost immediately to the consideration of the questions—

1. What are the objects of this operation?
2. What are its dangers?
3. To what particular cases is it applicable?
4. At what particular period of labour should it be performed?
5. What is the best method of performing the operation,—1, with regard to the instruments; 2, the particular part of the abdomen and uterus in which the incision should be made; 3, the manner of dressing the wounds through which the fœtus has been extracted?

The object of this operation is to extract a living child from the uterus or abdomen of a mother who has succumbed to some fatal accident previous to the delivery of the fruit of conception, whether this accident has occurred during labour, or some time previous to it. It has also for its object, to save the lives both of mother and child in those cases in which there are insuperable obstacles to the passage of the latter through the pelvis of the former. The necessity for this mode of delivery should of course depend upon the fact of the os uteri and other soft parts being in a state of great rigidity, or the pelvis so contracted as not to permit the ready exit of the fœtus through it, or, as relates to the child, of its situation in the uterus being such that its delivery could not be safely accomplished by the forceps, or by version and extraction by the feet.

Considering the circumstance that version by the feet is to be regarded as an operation always dangerous to the child, and that extraction by the head with the forceps in those cases in which there are no uterine or voluntary efforts on the part of the mother, must subject the fœtus to risk of injury, if not of immediate destruction of its life, we should regard delivery through the parietes of the abdomen not only the most expeditious, but also the most safe for the child.

When the death of the mother has ensued from rupture of the uterus, and the child is known to have passed into the cavity of the abdomen, there can be no hesitation as to the preference of gastrotomy over any other mode of delivery. The only question of importance involved is whether the child be still alive, and at how remote a period after the accident we might hope or reasonably expect to find its vitality preserved.

The existence of vitality in the child could perhaps be settled in many instances by auscultation, and although we might expect its death to occur in a short time after that of the mother, instances have been met with which prove that in some cases life is preserved several hours. Thus, Bordenave gives a case of the extraction of a living fœtus at sixth month of gestation, from the uterus of a woman who had died about six hours previously; and Gardien tells us of an instance in which a living child was removed, by the Cæsarean section, from the uterus of the mother, forty-eight hours after her death,—and further, that at the autopsy of the Princess Pauline of Schwarzemberg, who died of the effects of a scald at the fête of her brother-in-law, the Austrian ambassador at Paris, it was found that her fœtus was still alive, though her body was not opened until the day following.

The observations of Blundell in the few cases he has seen or collected, would tend to fix the limits of the vitality of the fœtus in utero at a much shorter period than those above mentioned. In the case in which his assistance was demanded, the abdomen of the woman was laid open thirteen minutes after the last respiration, and the child removed in two minutes more. By the use of proper means, the child shortly after began to respire.

Should auscultation fail to give any indications of the life of the fœtus, we think it would still be proper to resort to the operation for extraction, unless the period from the death of the mother had elapsed by several hours; and we think the operation would be especially indicated if the death of the mother had occurred suddenly, from any other cause than copious hæmorrhage; for although the sounds, and other evidences of the fœtal circulation, may at the time be absent, persevering efforts at resuscitation have often been successful in seemingly desperate cases. In the case under the notice of Mr. Tomkins, of Yeovil, the fœtus, after laying in a state of suspended animation more than an hour by his watch, was resuscitated by artificial respiration.

It will of course be of the utmost importance to determine the actual death of the pregnant woman before attempting an operation of so extensive a nature upon her person, as that intended for the removal of her child through the parietes of her uterus and abdomen. It is known that there are conditions of the system simulating death, from which the patient may completely recover.

In cases of great doubt as to the real occurrence of death in the mother, the question may properly be started, whether, since in case of real death, or the long continuance of suspended animation in the mother, the life of the fœtus is hazarded by any delay for the usual means of resuscitation, admitting that artificial delivery by the natural passages is impossible, are accoucheurs justified in proceeding at once to that operation which, while it gives a strong chance for the security of the life of the child, jeopardizes that of the mother?

Gastrotomy and gastro-hysterotomy have doubtless been performed on the female after death from the earliest periods of history, and as the operation involved no risk or suffering of the mother, and afforded a chance for the preservation of the child, no important obstacle opposed it under such circumstances. Notwithstanding the inference which may be drawn from the language found in the Nidda, a work regarded as an appendix to the Jewish Talmud, "that it is not necessary for the woman to observe the days of purification, after the removal of the child through the parietes of the abdomen," we might even suppose that the Cæsarean section had been practised upon the living female, and that she, in some cases, at least, recovered from the consequences of it; yet, we have no authentic cases of the performance of this operation upon the living female until about the year 1500, when Jacques Nufer, of Siegerhausen, opened the abdomen and uterus of his own wife after many fruitless attempts to deliver her through the natural passages, and thence extracted a living child, sewed up the wound, which united without any bad symptoms, and thus saved both his wife and child from imminent destruction.

Although this operation has had numerous opposers, some of whom have desired to substitute for it the use of the crotchet, and other instruments destructive of the life of the child, and more or less dangerous to the mother; and others have, without due consideration, highly recommended the Sigaultian operation, or symphysiotomy in all cases where the defects of the pelvis were so considerable as to render impracticable the passage of the child entire, or in a state of mutilation, through it, yet the operation of gastro-hysterotomy and gastrotomy has had its vindicators, and has been performed with somewhat variable success in different parts of the world, on the dead mother in early ages, and certainly on both living and dead since the bold and successful adventure of Nufer, about three hundred and fifty years ago. While Parè, Guillemeau, Marchand, Mauriceau, Sacombe, Osborn, and others, decidedly opposed the performance of this operation upon the living mother, Roussel, Weidmann, Bordenave, Baudelocque, and Hull, have collected numerous cases, which certify that the operation has been approved of and executed by numerous surgeons and accoucheurs of great respectability. Without entering here into the merits of the practice of the induction of artificial premature delivery, in certain cases of deformity of the pelvis, it may be sufficient for our present purpose to say that gastro-hysterotomy is positively demanded in those cases in which a woman arrives at term with a fœtus in utero, but with a pelvis so contracted, or with some other obstacle so insuperably interposed, as to render delivery impossible by the natural passages. It becomes a question of grave importance in case the child is living, and the diameters of the pelvis are so reduced as barely to admit of its passage in a state of infanticidal mutilation, and by such manipulation as must subject the mother to the hazard of her life.

The results of crotchet deliveries are so rarely the subjects of record, that statistics of them are very meagre, and we are therefore at a loss to compare them with what is known respecting the results of delivery through the parietes of the abdomen. One thing, however, is certain, that, abating the constant risk to which mothers are exposed, and the consequent death which some of them are known to suffer, no children are delivered by the crotchet in a state fit to live, however many of them, to the horror of the operator, may struggle for existence at the moment they are extracted, and while yet within the grasp of his fatal instrument.

We see, therefore, that while we consider obstetric operations in difficult cases, not amenable to the use of his forceps, restricted to the use of the perforator and crotchets, in all cases in which the fœtus is alive at the time of labour, the accoucheur deprives his country certainly of the prospective services of one citizen, at the same time that the parent is subjected to more or less risk of health, if not of life.

From the fact that we are unable to lay hold of the details of all the cases which have been reported in the collections by Hull, Baudelocque, and others, we are limited much within our wishes to make out such statistics as might contribute greatly to settle several questions of much interest to the community, as well as to the accoucheur. Dr. Hull, of Manchester, has collected accounts of one hundred and fifty-five cases previous to the year 1799. Of these a minute history is not always given, although some of them are narrated in considerable detail.

Of the women subjected to this operation, one hundred and twelve recovered, while forty-three others died.

Of the children of the women who recovered, forty-one are recorded to have been alive, while seven were saved, of the women who died. Many of the reports are silent on the subject of the condition of the child at the time of the operation.

Of seventy-three cases collected by Baudelocque, as having occurred between 1750 and 1800, thirty-one of the operations were performed with success to the mother, and almost all of them with success to the child; that amongst those women who could not be saved, there were several of whom no hopes were entertained before the operation, and others who appear to have been the victims of causes foreign to it, as sufficiently well-grounded hopes were entertained of them till the moment when these causes exerted their disastrous influence.

Simon relates sixty-four operations, more than one-half of which had been done on thirteen women. Some of these had undergone the operation once or twice; others, five or six times. There was one woman in particular who had undergone it seven times, and always with success.

If to these results, obtained entirely upon authority derived from the Eastern hemisphere, we add also what has been extracted by Bordenave from the Embryologia Sacra of Cangiamila, we shall greatly enlarge the amount of success, so far as relates to the preservation of the child, by stating that in the city and vicinity of Montreal, in the space of twenty-four years, twenty-one living children had been extracted by the Cæsarean operation: that between the year 1704 and 1748, sixty children had been extracted in the same manner at Caltanissecta, of whom only five were found dead: that at Victoria, a city in the diocese of Syracuse, between the years 1734 and 1752, the Cæsarean operation had been performed twenty times, and in every case a living child extracted; and that at Sambuca, a city in the diocese of Girenti, twenty-two pregnant women having died, from eighteen of them children were extracted alive.

If we add to these the cases collected by Dr. Nancrede, as having occurred in the United States, viz. one case of gastrotomy in 1792 in New York by Dr. M'Knight—one in Ohio by Dr. Richmond—one by Drs. Dougal and Vanvalrah in 1827, and another in 1832—one at Nassau, New York, by Drs. S. M'Clellan and Bassett, in the year 1823—several successful ones by Dr. Prevost of Louisiana, and on one patient several times—one by Dr. Nancrede in 1834, and again on the same patient under care of Drs. Fox and Meigs, in 1838, both of which last operations were completely successful; we shall have a large list of the cases of gastrotomy and gastro-hysterotomy, which have been recorded in the annals of the profession of medicine. Of the operations done in this country, I am not able to say that any of them have succeeded except perhaps those of Dr. Prevost, and certainly those of Nancrede and Fox.*

Having now seen that the Cæsarean section has been performed in a great many cases, in some of which it was doubtless the only expedient which could be resorted to with the view to effect delivery, and having seen that of those who have been the subjects of the operation, some die, and some escape, and become the subjects of subsequent operations of a similar kind, it is proper for us to inquire into the sources of the *danger* of this extensive abdominal incision.

The opponents of this operation seem to fear hæmorrhage, which they say must result from so extensive an incision into parts so vascular as some portions of the abdominal parietes, and particularly in that portion of the uterus with which the placenta has its peculiar connexion. Granting that the incisions may be made into such portions of the abdominal parietes as may be supplied by arterial branches of considerable magnitude, there could be little difficulty in securing them by the use of the needle and ligature.

Suppose, however, that the plexus of blood-vessels, both veins and arteries, which carry blood to and from that part of the uterus to which the placenta is attached, should lie so directly under the incisions just made, as to require that the operator cut through them in the division of the uterus; a free discharge of blood would of course be the immediate consequence; but as the child is soon extracted, and the uterus contracts, these vessels are supposed to be occluded, and rarely, if ever, to require a ligature. Is there not danger that the uterus may relax, and give out so much blood as to exhaust the patient, and that too by the secret effusion of the vital fluid into the cavity of the abdomen?

We think this might become a source of great danger, and even of fatality, to the sub-

* For an account of all that is known respecting the operation in this country up to the time that Mrs. Raybold, of this city, submitted to it, for the first time in 1834, see a very interesting paper in the American Journal of Medical Sciences, No. 32, August, 1835, by Dr. Nancrede; and for the report by Dr. Fox, of the 2d operation, see same periodical, No. 43, May, 1838.

ject of this operation. The testimony of most observers appears, however, to be that the hæmorrhage is small, rarely exceeding six or eight ounces, and scarcely ever more than that incident to natural labor.

Death rarely follows very soon after the operation, unless the patient be moribund at the time of its performance.

Since, then, the fatality of this operation does not appear to depend upon hæmorrhage, or on a severe shock to the nervous system causing tetanic convulsions, where are we to look for the destructive disorganization which results in the death of the whole animal machine ?

Surgeons are mostly inducted into practice with a dread of incising the serous investments of any cavity, whether articular or visceral, and hence would look with horror upon the longitude of one required for the delivery of a child through the abdominal parietes. Such was the insuperable dread which pervaded the mind of the father of American Surgery in this respect, that he persisted in his objections to the performance of the Cæsarean section in a case in which delivery by that mode was, in the opinion of many, most clearly indicated.

But is it the effects of an incision into the peritoneum which we are most to dread, and are we justifiable in allowing that fear to deter us from a prompt interposition, and an attempt to save the mother and child, when the latter is known to be alive, and its delivery through the natural passages, without mutilation, impossible?

How often is the peritoneal sac opened by accident, or by a surgical operation, and healed up without bad consequences ?

The researches of Blundell into the effects of wounds penetrating the cavity of the abdomen, have led him to the following presumptive inferences.

1. That smaller wounds of the peritoneum, as in tapping, hernia, &c., do not in general induce fatal peritonitis, or other destructive effects: and therefore the common opinion, not perhaps found on paper, but frequently urged in conversation, and apparently operative in practice, that inflammation in a spot of the peritoneum, will almost invariably diffuse itself over the greater part of it, is probably unfounded in truth.

2. That extensive divisions of the peritoneum are certainly not *of necessity* fatal, whether by inflammation or otherwise, and probably not generally so.

3. That the womb, spleen, and ovaries may be taken away, certainly without of necessity destroying life, and, *presumptively*, without generally destroying it.

4. That the womb, when developed from pregnancy, may be torn open; that the child may escape into the peritoneal sac, amongst the viscera; and that the mouth of the womb may be torn off—not, indeed, as far as these cases may be relied upon, without great danger, but twice in severe cases without death.

5. And, generally, that the peritoneum and abdominal viscera, though very tender in the human body, will, without fatal consequences, bear more injury, than, from their modes of practice, British (and, I may add, our older American) surgeons especially seem disposed to admit.

We may with great propriety inquire, as suggested by Denman, whether death is occasioned by any disease with which the patients were afflicted before the time of labour, or whether it is the consequence of the state to which the patients were reduced from the occurrence of labour before the operation was performed; or whether it be the inevitable consequence of the operation.

In reference to the unfortunate results of the operation in Great Britain, he remarks, that death happened at different periods, but not one died while being operated upon, or immediately after it. No convulsions were brought on by incisions, nor does it appear that any of them sunk through the loss of blood, accompanying or succeeding the operation.

Some died within twelve, others at the end of twenty-four hours, and a few on the third day after the operation. If we may judge of the cause of death from the period at which it occurred, it might be said that in those who failed within twenty-four hours, it was probably owing, not to the operation alone, but to the violence of this, combined with that of the previous disease; but when they survived twenty-four or forty-eight hours, their death might be attributed to the succeeding inflammation in a body previously disposed to disease.

If we had the liberty of selecting a patient on whom to try the merits of this operation, we certainly should not choose one who was either very much distorted, or who had the mollities ossium, or who was evidently under the influence of some dangerous disease, or who had been several days in labor, because the event must very much depend upon the state of the patient at the time of the operation.

While we are ready to make a general concession to the views of Denman, and are apprehensive with Blundell that in many cases of death after the Cæsarean section, have depended upon a cachectic state of the system affected with mollitis ossium, yet, if we make an analysis of the cases furnished by Hall, we shall find several instances of complete recovery after the patient had been subjected to severe but unavailing contractions of the uterus when the operation was attempted. Thus in a table of 110 successful operations, it is ascertained by the accompanying reports, that one patient had been in labour two days; three, three days; four, four days; one, five days; one, seven days; two, *several days*, and one "very long."

We are told also that before Nufer operated on his own wife she had been several days in labour, and many fruitless attempts had been made to deliver her, so that she must have been in a state of much excitement, and apparent unfitness for the operation, yet not only she recovered but her child was removed alive. In the case in which the labour had continued seven days, the patient had been subjected to the unsuccessful use of the crotchet. Her recovery however was without accident.

In the case of Alice O'Neil, of Ireland, operated upon by Mary Donnally, the patient had been in labour twelve days, yet she was able to walk from home on the 27th day after the unsurgical operation to which she had been subjected.

Jane Foster, upon whom gastrotomy was performed in consequence of rupture of the uterus, from distortion of the pelvis, after having been in labour *five* days had the wound healed in six days, and was able to resume her usual employment at the end of twenty days.

Several of the successsful cases enumerated in Dr. Hull's collection, had been subjected to the Cæsarean section in consequence of pelvic deformity from ricketts.

These cases serve to show that success sometimes accompanies the operation, even under very unpromising circumstances.

As very little blood is usually lost by this operation ; as few or no instances are known of convulsions arising from a shock to the nervous system by so extensive an incision into the abdomen, we may ask what is the source of danger in this operation ?

It appears to be pretty generally agreed, that in cases which terminate fatally, if the patient do not speedily sink from exhaustion induced by the previous disease or the severity and duration of her labour, added to the shock of so extensive an incision, she rarely succumbs until the peritoneum becomes extensively inflamed. And it is perhaps this diffused inflammation of the peritoneum which is to be most dreaded.

How can the operator guard against the risk of the occurrence of this accident ?

Without knowing precisely what stimulus it is, which excites this inflammation, when the operation has been performed skilfully and with sharp insruments, some surgeons have attributed it to the action of atmospheric air, or at least to its contact with this sensative membrane at a reduced temperature. Mr. Lizars, a surgeon of considerable experience in abdominal wounds, whether accidental, or designed, gave as his opinion that, with a view to obviate the dangers, from the exposure of the peritoneum to an atmosphere of depressed temperature, the apartment in which the operation was to be performed should be warmed to above 90 degrees of Farenheit's thermometer, and hence not many degrees below the temperature of the internal parts of the body. In order completely to avoid the contact of atmospheric air with the peritoneal surface, Dr. Aitkin inquires, "whether the performance of this operation under the surface of the tepid water, by secluding the atmospheric air, would not tend to diminish it fatal effects."

Some experiments by Dr. Haighton on this point would seem to settle this question negatively. He opened the abdomen of different animals to a considerable extent, closing them during the immersion, and applying to the cut edges of the abdominal wound, as soon as it was raised above the water, an adhesive composition, by which, and a bandage, the external air was excluded.

"No symptoms indicating internal inflammation supervened, and perfect health was soon restored. After the lapse of some weeks, these animals were subjected to anatomical examination, which showed that *an adhesion of the omentum* to the cut edges of the peritoneum had made that cavity complete." Dr. Hull says, Haighton afterwards repeated these experiments in the open air, all the other circumstances being similar, and the event of them agreed precisely with the former. Not content with this mode of experimenting, he reversed it by injecting air with a syringe into the cavity of the peritoneum of dogs, introducing it through the tumica vaginalis testis. "From different trials which were made in this manner," he says, "I do not hesitate to conclude that atmospherical air, penetrating into cavities, is perfectly harmless and has no influence over the consequences of the Cæsarean operation."

Allowing the merits of these experiments, observations and opinions, and conceding that while some females have recovered from the operation who have been horribly deformed by ricketts and worn down by a tedious and unavailing labour of several days, and that the abdominal and uterine incisions have in some of these cases been made after every other manipulation, and even thd crotchet had been resorted to without effect, while other patients have died in a short time after this operation, although they were previously in good health and had been in labour little more than 24 hours, we may very naturally inquire whether the result of the case depends as much upon any peculiar skill in the operator as upon the great tenacity of life which some constitutions appear to possess.

Some very striking instances of recovery have occurred under circumstances apparently unfavourable, and with what might be regarded, if not bad, certainly very indifferent surgery. Of the former class may be noticed the case of —— Brument, in the district of Candebec, in the department of the Lower Seine, æt. thirty-nine, mother of several children, in the eighth month of her pregnancy. She received a blow from the horn of a bull in July, 1789, which opened the hypogastric region transversely, and the anterior part of the womb to the extent of more than ten inches. She was in the fields at the time at considerable distance from home. The child immediately passed through this extensive laceration, which was attended with a prodigious loss of blood. The persons present being alarmed by her situation, durst not attempt to relieve her before the arrival of Lechaptois, a surgeon a league and a half distant, and contented themselves with wrapping the infant, which was alive, in an apron, without dividing the navel string,—so that more than an hour and a half passed before she obtained any assistance. Lechaptois began by dividing the funis imbilicalis to separate the child from the mother, who was in such a feeble state that her pulse could scarcely be perceived. He then extracted the placenta through the wound, the womb being contracted and the hæmorrhage restrained. He afterwards cleaned the intestines and the adjacent parts, which were covered with blood and earth, by means of a sponge moistened in wine and water, replaced them, made eight stitches of the quilled suture, and covered the whole with a suitable dressing and bandage. The woman was then carried home. The child lived eight hours. The woman in six weeks was completely cured. Five years afterwards she enjoyed very good health, and has only suffered from a hernia in the part where two of the stitches had divided the lips of the wound.

There are on record at least two cases of self-operation by the Cæsarean section, one a negro woman in Jamaica, who, in 1769, tired out with the pains of labour, took a broken butcher knife, about two inches and a half of which remained attached to the handle, and with this made an incision in her left side, near the linea alba, and so deeply as to wound the right thigh of the child, who escaped through the abdominal opening by its own struggles, followed by a considerable portion of the intestines, which were pushed back by a negro midwife, who was sent for to her assistance. After a few hours had elapsed, the surgeon of the plantation, supposing the old woman had left some dirt in the wound, cut open the stitches that had been made, carefully washed the parts clean, extracted the placenta at the wound, and then stitched it up again. This woman was able to attend to her work in six weeks.

A mulatto girl, residing at Nassau, delivered herself in 1823, of twins, by making an incision through the parietes of the abdomen and uterus, and recovered from her wounds, which were dressed by Drs. S. M'Clellan and Bassett.

The first case of Cæsarean section which the records of Great Britain afford us, was performed by Mary Donnally, a midwife, on the person of Alice O'Neil, near Charlemont, in Ireland. This intrepid *chirurgiene-accoucheuse* made her incision with a razor, extracted the child, and held the wound while a messenger was despatched a mile distant for some silk and tailor's needles, with which she sewed up the wound, and then smeared it with whites of egg. This Alice O'Neal recovered in twenty-seven days. Other cases could be cited, tending to show that some women recover from the operation, although little claim can be laid to great surgical skill in the performance of it.

We have already stated that the want of correct statistics of the results to the mother of embryulcia deprives us of the opportunity of making any comparison of the value of the Cæsarean section, and embryotomy in certain cases of impracticable labour. We are, however, strongly inclined to the belief that, could the catalogues of disasters in both modes of delivery be spread fully before us, the preponderance of benefits would be less in favour of the crotchet, admitting the child to be alive at the time of the operation, than we have hitherto supposed.

